# Gene conversion and purifying selection shape nucleotide variation in gibbon L/M opsin genes

**DOI:** 10.1186/1471-2148-11-312

**Published:** 2011-10-22

**Authors:** Tomohide Hiwatashi, Akichika Mikami, Takafumi Katsumura, Bambang Suryobroto, Dyah Perwitasari-Farajallah, Suchinda Malaivijitnond, Boripat Siriaroonrat, Hiroki Oota, Shunji Goto, Shoji Kawamura

**Affiliations:** 1 Department of Integrated Biosciences, Graduate School of Frontier Sciences, The University of Tokyo, Kashiwa 277-8562, Japan; 2 Department of Behavioral and Brain Sciences, Primate Research Institute, Kanrin, Inuyama, Aichi 484-8506, Japan; 3 Department of Biology, Faculty of Mathematics and Natural Sciences, Bogor Agricultural University, Kampus IPB Darmaga, Bogor 16680, Indonesia; 4 Primate Research Center, Bogor Agricultural University, Jalan Lodaya II/5, Bogor 16151, Indonesia; 5 Primate Research Unit, Department of Biology, Faculty of Science, Chulalongkorn University, Bangkok 10330, Thailand; 6 Research and Conservation Division; Zoological Park Organization (ZPO), Bangkok, Thailand; 7 Center for Human Evolution Modeling Research, Primate Research Institute, Kanrin, Inuyama, Aichi 484-8506, Japan; 8 Department of Rehabilitaion, Chubu Gakuin University, Kirigaoka 2-1, Seki, Gifu 501-3993, Japan; 9 Department of Anatomy, Kitasato University School of Medicine, Kitasato 1-15-1, Sagamihara, Kanagawa 252-0374, Japan; 10 Faculty of Veterinary Medicine, Azabu University, Fuchinobe 1-17-71, Sagamihara, Kanagawa 252-5301 Japan

## Abstract

**Background:**

Routine trichromatic color vision is a characteristic feature of catarrhines (humans, apes and Old World monkeys). This is enabled by L and M opsin genes arrayed on the X chromosome and an autosomal S opsin gene. In non-human catarrhines, genetic variation affecting the color vision phenotype is reported to be absent or rare in both L and M opsin genes, despite the suggestion that gene conversion has homogenized the two genes. However, nucleotide variation of both introns and exons among catarrhines has only been examined in detail for the L opsin gene of humans and chimpanzees. In the present study, we examined the nucleotide variation of gibbon (Catarrhini, Hylobatidae) L and M opsin genes. Specifically, we focused on the 3.6~3.9-kb region that encompasses the centrally located exon 3 through exon 5, which encode the amino acid sites functional for the spectral tuning of the genes.

**Results:**

Among 152 individuals representing three genera (*Hylobates*, *Nomascus *and *Symphalangus*), all had both L and M opsin genes and no L/M hybrid genes. Among 94 individuals subjected to the detailed DNA sequencing, the nucleotide divergence between L and M opsin genes in the exons was significantly higher than the divergence in introns in each species. The ratio of the inter-LM divergence to the intra-L/M polymorphism was significantly lower in the introns than that in synonymous sites. When we reconstructed the phylogenetic tree using the exon sequences, the L/M gene duplication was placed in the common ancestor of catarrhines, whereas when intron sequences were used, the gene duplications appeared multiple times in different species. Using the GENECONV program, we also detected that tracts of gene conversions between L and M opsin genes occurred mostly within the intron regions.

**Conclusions:**

These results indicate the historical accumulation of gene conversions between L and M opsin genes in the introns in gibbons. Our study provides further support for the homogenizing role of gene conversion between the L and M opsin genes and for the purifying selection against such homogenization in the central exons to maintain the spectral difference between L and M opsins in non-human catarrhines.

## Background

In catarrhine primates (humans, apes and Old World monkeys) the L and M opsin genes are closely juxtaposed on the X chromosome and, in combination with the autosomal S opsin gene, enable routinely trichromatic color vision [1,2]. The L and M opsin genes have a close evolutionary relationship and are highly similar in nucleotide sequence (~96% identity). Among 15 amino acid differences between the human L and M opsin genes, three account for the main shifts in spectral sensitivities and tuning [3-9].

The organization of the L and M opsin genes among humans is known to be variable and includes the absence of an L or M opsin gene or the presence of L/M hybrid genes with an intermediate spectral sensitivity. A high incidence (approximately 3-8%) of color vision "deficiencies" in males results as a consequence [10]. This variation is caused by unequal meiotic recombination between L and M opsin genes. When the nucleotide sequence of a part of one gene appears to be replaced with the corresponding sequence of the other, this type of recombination is often called gene conversion. Gene conversion is suggested to have occurred frequently between the human L and M opsin genes [11-15] and to have played a crucial role in generating hybrids of the two genes with altered spectral sensitivities [16-18]. Even among individuals with normal color vision, the allele frequency of the L opsin gene with Ala at the site 180 instead of Ser is reported to be 30-38% in non-African populations [16-18].

Compared to humans, the incidence of color vision variation is reported to be rare in other catarrhines [19-21]. Among 744 male long-tailed macaques (*Macaca fascicularis*) examined, only three were found to have a single L/M hybrid gene with an intermediate spectral sensitivity and to be dichromats [19,22,23]. Among 58 male chimpanzees (*Pan troglodytes*), one was found to have an L/M hybrid gene with an intermediate spectral sensitivity in addition to one normal M opsin gene on the X chromosome and to be a protanomalous trichromat [21,24]. Thus, frequencies of color vision variants in male long-tailed macaques and male chimpanzees can be calculated to be ~0.4% and ~1.7%, respectively. These frequencies could be overestimated because no variants were found in 455 male monkeys from other macaque species [19,23] and because the chimpanzees examined were from limited numbers of breeding colonies [21]. Other studies have reported an absence of color vision defects in Old World monkeys and apes [20,25].

Nevertheless, gene conversion is suggested to have occurred frequently between the L and M opsin genes in non-human catarrhines on the basis of the following observations: (1) the intraspecific nucleotide divergence between the L and M opsin genes (paralogous divergence) tends to be smaller than divergence of the same gene between species (orthologous divergence) [26,27], (2) allelic polymorphism is often shared between L and M opsin genes and between species [28,29], (3) paralogous nucleotide divergence in introns and peripheral exons (exons 1 and 6) is significantly smaller than those in the centrally located exons (exons 2-5), which contain the amino acid sites affecting absorption spectra of the L and M photopigments [13,14]. These studies suggest that gene conversions at nucleotide sites relevant for the spectral difference between the L and M opsins have been effectively eliminated from the population by purifying natural selection.

If gene conversion occurred frequently between the L and M opsin genes and if purifying selection was active in non-human catarrhines, we would also expect another intraspecific pattern of nucleotide variation: higher nucleotide divergence between the L and M opsin genes in central exons than in introns in addition to lower nucleotide diversity within these exons than within introns. However, the within-species nucleotide variation of both exons and introns has been evaluated for only the L opsin gene of two African hominoids, humans [18] and chimpanzees (primarily *P. t. verus*) [25]. In the present study, we focused on gibbons (Family Hylobatidae), commonly known as the lesser apes, for which normal trichromacy is reported [30]. Gibbons occur in Asia and are the most diverse and speciose of all living apes [31], making them an ideal group with which to assess the range of L/M opsin genetic variation. We examined the nucleotide variation of both the L and M opsin genes by sequencing the 3.6~3.9-kb genomic region encompassing exon 3 to exon 5 from individuals in five species and three genera of gibbons.

## Methods

### Gibbon DNA samples

Blood samples were collected from a total of 157 individuals of the following species: Agile (*Hylobates agilis*; N = 37), Kloss' (*H. klossii*; N = 2), White-handed (*H. lar*; N = 40), Silvery Javan (*H. moloch*; N = 6), Mueller's Bornean gray (*H. muelleri*; N = 6), Pileated (*H. pileatus*; N = 19), Chinese White-cheeked (*Nomascus leucogenys*; N = 16) and Siamang (*Symphalangus syndactylus*; N = 31). Sampling was conducted at the Ragunan Zoo and the Pontianak Zoo in Indonesia, and the Chiang Mai Zoo, the Bangkok Zoo and the Khao Kheow Open Zoo in Thailand. We also sampled gibbons reared by local residents in Kalimantan, Indonesia. Genomic DNA was extracted from blood samples using the DNA Microextraction Kit (Stratagene, Santa Clara, CA) or the QIAamp DNA Blood Mini Kit (Qiagen, Duesseldorf, Germany). Research permissions were granted by each country and sampling was conducted according to the Guide for the Care and Use of Laboratory Animals by the National Institute of Health, U.S.A. (1985) and the Guide for the Care and Use of Laboratory Primates by the Primate Research Institute, Kyoto University (1986, 2002). All procedures were approved by the animal ethics committee of the Primate Research Institute, Kyoto University.

Among the 157 individuals, 152 were subjected to the genotyping of the L/M opsin genes (Additional file 1, Table S1). The remaining 5 individuals (two *H. agilis*, one *H. lar*, and two *S. syndactylus*) were included in the analysis of the neutral reference genes. Among the 152 individuals, 94 were subjected to DNA sequencing of the entire 3.6~3.9-kb region encompassing exon 3 to exon 5 (Table 1).

**Table 1:** The number of gibbon individuals for which the L and M opsin genes were sequenced

Species name	Male	Female	Total
*Hylobates agilis*	12	8	20
*H. lar*	14	10	24
*H. pileatus*	10	6	16
*Nomascus leucogenys*	5	8	13
*Symphalangus syndactylus*	10	11	21

Total	51	43	94

### Genotyping and sequencing of the gibbon L and M opsin genes

In primates, the L and M opsin genes are arrayed in the same orientation on the X-chromosome and separated by approximately 24 kb [32]. Both genes consist of six exons that encode a protein 364 amino acids long, which spans approximately 15 kb [1,33,34]. The first (most upstream) position in the gene array is typically occupied by the L opsin gene, followed by one or more M opsin genes downstream [35,36]. However, the long-range polymerase-chain reaction (PCR) necessary to determine the position of an L or M opsin gene in the array is not feasible for a large number of samples due to the paucity of gene-specific nucleotide sites and of gene-position specific nucleotide sites [21,37-40]. We thus identified a gene not by the position in the gene array, but rather by its inferred spectral property.

The peak absorption spectra (λmax) of the catarrhine L and M opsin photopigments are approximately 560 nm and 530 nm, respectively [41]. This spectral difference is mainly attributed to the amino acid differences at three sites: the residue 180 encoded in the exon 3 and the residues 277 and 285 in the exon 5 [3,4]. Amino acid changes from Ser to Ala at the site 180, (denoted Ser180Ala), Tyr277Phe, and Thr285Ala shift the λmax values by -7, -8, and -15 nm respectively, in nearly an additive manner, and the reverse amino acid changes cause opposite spectral shifts by approximately the same extent [5-8]. Therefore, the contribution of exon 5 to the difference of absorption spectra between the L and M opsins is greater than that of exon 3.

Based on the inferred absorption spectra, we defined the gibbon L opsin gene as having Tyr and Thr and the M opsin gene as having Phe and Ala at the residues 277 and 285, respectively. This differs slightly from previous studies in which gene identity is also based on other nucleotide differences with little or no spectral effects [16]. We defined the spectral hybrid as having Ala at the reside 180 in the exon 3 of the L opsin gene and as having Ser at the exon 3 of the M opsin gene.

The average size of the amplified region among the M opsin sequences was approximately 3.6 kb in each species, while that of the L opsin gene was approximately 3.9, 3.8 and 3.6 kb in the *Hylobates *species, *N. leucogenys *and *S. syndactylus*, respectively. The size difference of the genomic region between the L and M opsin genes was due to an Alu element in the intron 3 of the L opsin gene. We isolated this genomic region by two sets of PCR schemes.

For the first PCR set, we designed an L-specific and an M-specific reverse primer, Ex5L_Rev (5'- CCCCAGCAGACGCAGTACGCAAAGAT -3') and Ex5M_Rev (5'- CCCCAGCAGAAGCAGAATGCCAGGAC -3'), at a region including residue 277 and five surrounding nucleotide sites at which the human L (GenBank Accession number Z68193) and M (GenBank AC092402) opsin genes differ. This primer site does not contain the residue 285, which is attributable to the relatively few nucleotide differences reported between L and M immediately downstream of residue 285. Combined with this, a forward primer common to the human L and M opsin genes was set in intron 2, just upstream of exon 3 (Ex3_For: 5'-GGATCACAGGTCTCTGGTCTCTG-3'). The nucleotide sequence of exon 3 was then determined for the PCR products using a sequencing primer common to the L and M opsin genes (Ex3_Rev: 5'-GAGCGTGCAATGTCTATCAA-3') located in intron 3. The nucleotide differences in the region used for the gene-specific reverse primers and in the residue 285 downstream of the primers in the exon 5 were confirmed by a second PCR set.

Our second PCR set was comprised of a reverse primer common to the human L and M opsin genes. It was designed in intron 5 just downstream of exon 5 (Ex5_Rev: 5'-GAAATGACCGGGAAAGGCTC-3'). An L- and an M-specific forward primer were designed in exon 3 at a region upstream of the site 180, Ex3L_For (5'-TTTCCTGGGAGAGGTGGCTGGTGGTG-3') and Ex3M_For (5'-TTTCCTGGGAGAGATGGATGGTGGTC-3'), so as to include three differing nucleotide sites between the published human L and M opsin genes. The nucleotide sequence of exon 5 was then determined for the PCR product using a common sequencing primer between the L and M opsin genes (Ex5_For: 5'-CACTCAGGGCTGGAAGATGGC-3') designed in intron 4. The nucleotide differences in the region used for the gene-specific forward primers in exon 3 were confirmed by the first PCR set described above.

By using the combination of these two PCR sets, we avoided the possible misinterpretation of the presence or absence of a gene type by false-positive or false-negative amplification by the gene-specific primers. This is an improvement from previous studies relying on the success of PCR using gene-specific primers [16,21].

PCRs were carried out in 25 μl containing 1.5 units of Ex Taq polymerase hot start version (Takara, Tokyo) with 1× Ex Taq Buffer, 0.2 mM each of dNTPs, 1 μM each of the forward and reverse primers, and ca. 5 ng of the gibbon genomic DNA at 94°C for 10 min followed by 35 cycles at 94°C for 30 sec, 65°C for 30 sec, and 72°C for 4 min. Distilled water was used as the template for the negative control in every reaction. The amplification was confirmed by 0.5% agarose-gel electrophoresis and the amplified DNA fragments were purified using the Montage PCR Centrifugal Filter Device (Millipore, Tokyo).

Pruified DNA samples were directly sequenced using Applied Biosystems model 3130 automatic sequencer with Big Dye Terminator v3.1 Cycle Sequencing kit (Applied Biosystems Japan, Tokyo) and sequencing primers (Ex3_Rev, Ex5_For and other primers set inside the amplified region). Operationally, we first sequenced the L and M opsin genes of both DNA strands from a sample of a male as only one X-chromosome is present. We sequenced only one strand for other samples if there was no nucleotide difference from the ones for which both strands were sequenced. When we found nucleotide differences in two or more individuals at the same site, we sequenced both strands of at least one of the samples to confirm the variation. When we found a different nucleotide in only one sample, *i.e*. as a singleton among the samples, we repeated the PCR for the sample and sequenced the region for both strands to rule out a PCR error and to confirm the variation.

When a sample from a male showed two nucleotide peaks at one or more sites ("heterozygous" sites) in the L or M opsin gene, the individual should have two or more loci of the gene with different sequences. In this case, we conservatively inferred two loci for this gene. When a female showed heterozygous sites, the individual could have allelic differences or two or more copies of the gene. We regarded such females as having one locus with different alleles for this opsin type because this arrangement gives the minimum possible and the most conservative estimate of the number of sequences. Thus, the sequence number could be underrepresented in our results and one gene group (*i.e*. the L or M opsin group) does not necessarily consist of orthologous members. When we confined an analysis to the orthologous gene groups, we used only male samples with single L and M opsin genes.

We did not attempt to separate the sequences by DNA cloning. In the alignment of the nucleotide sequences (Additional files 2, 3, 4, 5 and 6), we treated these sequences as two alternate gene sequences and arbitrarily assigned the nucleotides at the heterozygous sites into the two sequences. For calculations of the nucleotide diversity within a gene group and the nucleotide divergence between gene groups (see below for details), the nucleotide combination at the heterozygous sites (haplotype) is not necessary. When we conducted an analysis requiring the haplotype information, such as the GENECONV procedure (described below) or the evaluation of linkage disequilibrium, we used only male samples with single L and M opsin genes.

### Nucleotide sequencing of the neutral reference regions

The eta-globin gene is suitable as a neutral reference because it is a single copy pseudogene well characterized for primates [42] and the ortholog for a gibbon (*H. lar*) has been reported (GenBank M54985) [43]. A pair of PCR primers was designed according to the gibbon sequence to amplify ca. 0.5 kb in the 5'-flanking region that is free of repeat sequences: forward, 5'-AGAGGAGAAGTAAAAAGCCAC-3'; reverse, 5'-ACATAACTCTCAAAATCCCAC-3'. The S opsin gene intron 4 was also used as a neutral reference. A pair of PCR primers was designed according to the human S opsin sequence (GenBank L32835) to amplify ca. 0.5 kb that is free of repeat sequences: forward, 5'-CCCTGCCAACTTTTAGCTTGCAC-3'; reverse, 5'-TTCCCGCACCATCTCCATGAT-3'.

The PCRs were carried out with the same composition of the reaction mixture as used for the L/M opsin gene. Purification and sequencing were carried out as previously described for the L/M opsin gene. As was the case with the L/M opsin gene, we did not clone the neutral references and thus the two allelic DNA sequences of individuals were not separated. In the alignment (Additional files 7, 8), two heterozygous alleles were tentatively sorted into two allele sequences.

In each of the five gibbon species, the nucleotide sequences of the L and M opsin genes from one male sample with single L and M opsin genes have been deposited to the GenBank/EMBL/DDBJ database. The nucleotide sequences of the eta-globin 5' flanking and the S opsin intron 4 sequences were also deposited to the database from one individual each from the five species who had no heterozygous sites. Their sequence ID in this study (see also Additional files 2, 7, 8) and the accession numbers in the database are listed in Table 2.

**Table 2:** The GenBank/EMBL/DDBJ accession numbers of gibbon L and M opsin genes

Species name	ID	Accession number
*Hylobates agilis*		
L	1_Hag_M_L	AB670154
M	1_Hag_M_M	AB670155
eta	1_Hag_E_1	AB670164
S	1_Hag_S_1	AB670169

*H. lar*		
L	132_Hla_M_L	AB670156
M	132_Hla_M_M	AB670157
eta	55_Hla_E_1	AB670165
S	23_Hla_S_1	AB670170

*H. pileatus*		
L	29_Hpi_M_L	AB670158
M	29_Hpi_M_M	AB670159
eta	30_Hpi_E_1	AB670166
S	28_Hpi_S_1	AB670171

*Nomascus leucogenys*		
L	107_Nle_M_L	AB670160
M	107_Nle_M_M	AB670161
eta	115_Nle_E_1	AB670167
S	111_Nle_S_1	AB670172

*Symphalangus syndactylus*		
L	6_Ssy_M_L	AB670162
M	6_Ssy_M_M	AB670163
eta	4_Ssy_E_1	AB670168
S	3_Ssy_S_1	AB670173

### Data analysis

Alignment of the nucleotide sequences was conducted using the Clustal W software [44] and was adjusted manually. The average number of between-group nucleotide differences per nucleotide site was designated the nucleotide divergence (*d*). The *d *value between the L and M opsin genes within a species was calculated by eliminating all positions containing gaps from the alignment. The average number of within-group nucleotide differences per nucleotide site between two sequences was designated the nucleotide diversity (π) and was calculated by eliminating all positions containing gaps from the alignment. The average numbers of between- and within-group synonymous differences per synonymous site and non-synonymous differences per non-synonymous site were calculated using the Nei-Gojobori method [45]. We connected the alignments of the eta-globin pseudogene and the S opsin gene intron 4 to calculate the π value of the combined neutral references for the individuals for which both genes were sequenced. The standard errors of the π and *d *values including those of their synonymous and non-synonymous components were estimated by a bootstrap procedure (1000 replicates). All of these computations were conducted using MEGA version 5 [46]. The statistical significance of the difference between two *d *value or two π values was evaluated by the one-tailed Z test.

For constructing an among-group phylogenetic tree, we corrected the *d *values for multiple substitutions by the Jukes-Cantor formula [47] and used the neighbor-joining method [48] by eliminating all positions containing gaps from the alignment. The exon and intron sequences of a human L (GenBank Z68193) and M (GenBank AC092402) opsin genes and the exon sequence of a crab-eating macaque L (GenBank AF158968) and M (GenBank AF158975) opsin genes [23] were also included as well as the exon sequence of a mouse M opsin gene (GenBank AF011389) as an outgroup. In the case of the human, macaque and mouse sequences, one gene sequence was regarded as one group. The reliability of the topology of the phylogenetic tree was evaluated by bootstrap resampling (1000x) using the "sendbs2" program [49]. To compute the bootstrap values, the human, macaque and mouse sequences were input twice in the alignment because the sendbs2 does not accept one sequence as one group.

For testing gene conversion, we applied the GENECONV 1.81 program [50] implementing Sawyer's statistical method [51]. GENECONV searches for a consecutively identical region between two sequences and evaluates the probability of achieving homogeneity when nucleotide differences are assumed to be randomly distributed throughout the sequence region.

## Results

### L/M opsin genotyping

On the basis of the amino acid composition at site 180 encoded in the exon 3 and 277 and 285 encoded in exon 5, the L/M opsin genotype was determined for 152 individuals of gibbons from the eight species (Additional file 1, Table S1). All individuals had standard L and M opsin genes. No L/M hybrid genes were found in terms of the three-site composition.

### Multiple M opsin copies

The nucleotide variation was analyzed for five species, *H. agilis*, *H. lar, H. pileatus*, *N. leucogenys *and *S. syndactylus *for the genomic region encompassing the exon 3 through the exon 5, approximately 3.6~3.9 kb. We excluded *H. klossii*, *H. moloch *and *H. muelleri *from the subsequent analysis because of their small sample size. A total of 94 individuals including 51 males were subjected for the analysis (Table 1). Among the males, there were no individuals detected that had multiple L opsin genes. On the other hand, multiple M opsin genes were detected in 80.0% of *N. leucogenys*, 28.6% of *H. lar*, 16.7% of *H. agilis*, and 10.0% of *H. pileatus *and *S. syndactylus *males.

### Nucleotide divergence between L and M opsin genes within species

**Figure 1:**
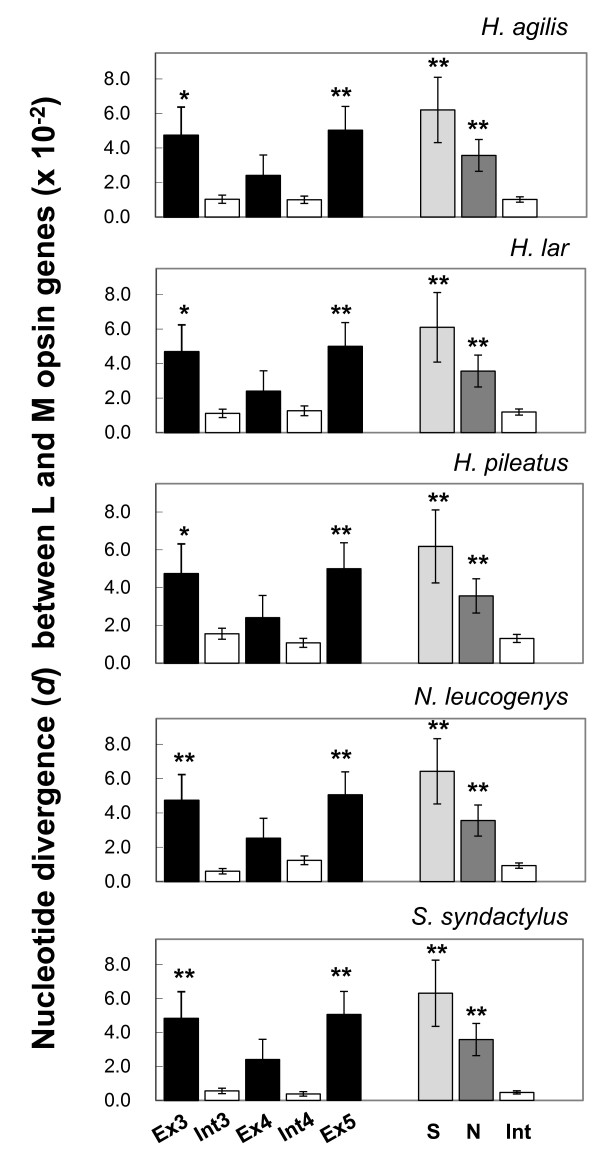
**The nucleotide divergence (*d*) between the L and M opsin genes in five species of gibbons, *H. agilis*, *H. lar*, *H. pileatus*, *N. leucogenys *and *S. syndactylus***. The *d *values of the exons are represented by black, the introns by white, the synonymous sites by light gray, and the non-synonymous sites by dark gray bars. The error bars indicate the estimated standard error of the *d *values based on the 1000 × bootstrap resampling. The asterisks indicate that the *d *values are significantly higher than the *d *value of the combined sequence of the introns 3 and 4 in each species. The single and double asterisks represent the statistical significance at 0.05 and 0.01 levels, respectively, based on the one-tailed Z test. Ex3, exon 3; Ex4, exon 4; Ex5, exon 5, Int3, intron 3; Int4, intron 4; Int, the introns 3 and 4 combined; S, synonymous sites in the exons 3, 4 and 5; N, non-synonymous sites in the exons 3, 4 and 5.

Figure 1 summarizes the nucleotide divergence between the L and M opsin genes in the exons and introns in the five gibbon species (Additional file 1, Table S2 for the values). Exons were generally more divergent than introns. In particular, the divergence of exons 3 and 5 was significantly higher than that in the introns. Within the exons, however, non-synonymous divergence was lower or not significantly higher than synonymous divergence in any of the three exons in all five species (Additional file 1, Table S3). Both synonymous and non-synonymous divergences of exons were significantly larger than the intron divergence in all five species (Figure 1).

We also examined male samples with a single M as well as a single L opsin sequence types. There were ten *H. agilis*, ten *H. lar*, nine *H. pileatus*, one *N. leucogenys*, and nine *S. syndactylus *males that had single L and M opsin genes. We excluded the one *N. leucogenys *from the calculation because of its inapplicability to the population analysis. As shown in Additional file 1, Figure S1, the pattern of nucleotide divergence between the L and M opsin genes in the exons and introns in these individuals was essentially the same as the pattern shown in Figure 1.

### Nucleotide diversity of L and M opsin genes within species

**Figure 2:**
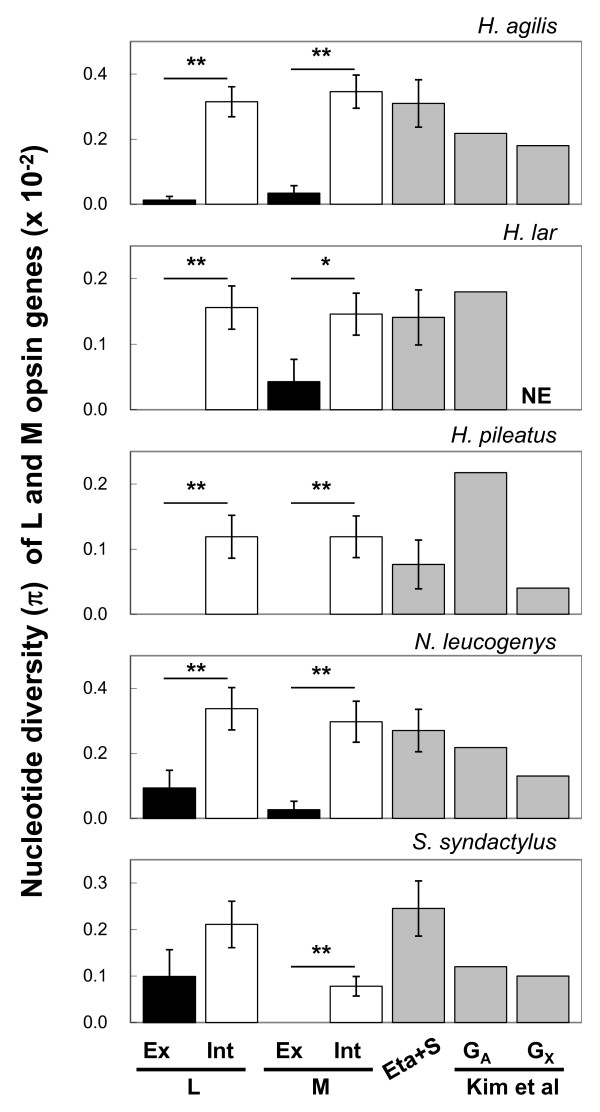
**The nucleotide diversity (π) of the exons (black bar) and the introns (white bar) of the L and M opsin genes and the neutral references (gray bars) in the five species of gibbons**. The gibbon genomic data reported for autosomal (G_A_) and X-chromosomal (G_X_) regions [52] are also indicated as neutral references. The π values of the combined sequences of the eta globin pseudogene and the S opsin intron 4 (Eta+S) and the π values of G_A _are multiplied by 3/4. The single and double asterisks represent the statistical significance at 0.05 and 0.01 levels, respectively, based on the one-tailed Z test.

Figure 2 summarizes the nucleotide diversity of the L and M opsin exons and introns and of the neutral references (see Additional file 1, Tables S4 and S5 for the values). As for the neutral references, we also incorporated the nucleotide diversity reported by Kim et al (2011) [52] for the30~75 kb of autosomal regions and the 2~13 kb of X chromosomal regions of these gibbon species. In Figure 2, the nucleotide diversity values of the autosomal neutral references are adjusted (multiplied by 3/4) for comparison with X-chromosomal L/M opsin genes (see Discussion).

In both the L and M opsin genes, contrary to the case with the nucleotide divergence, the nucleotide diversity of the exons was lower overall than that of the introns and in many cases was even zero (Additional file 1, Table S4). Alternatively, introns were as variable as the neutral references (Figure 2). When the three exons and the two introns were combined respectively, the exons were significantly less variable than the introns (Figure 2). When using only males with single L and M opsin genes, the pattern of nucleotide diversity of L and M opsin genes (Additional file 1, Figure S2) was also the same with that shown in Figure 2.

Within the exons, variation consisted mainly of synonymous variation (Additional file 1, Figure S3 and Table S6). The synonymous variation was as high as, or lower than, the intron variation. However, the synonymous variation showed a large stochastic error compared to the nucleotide diversity in the introns (Figure S3) and the synonymous nucleotide divergence (Figure 1). This is because of the much smaller number of the synonymous nucleotide sites (137~138 bp) in the coding region than in the total length of the introns (3.0~3.3 kb) and the lower level of synonymous nucleotide diversity (0~0.4%) than the synonymous divergence between the L and M opsin genes (6.1~6.4%).

### Comparison of inter-LM divergence and intra-L/M polymorphism between synonymous sites and introns

Using the male samples with single L and M opsin genes, we counted the number of sites fixed with different nucleotides between the L and M opsin genes (inter-LM divergence) and the number of polymorphic nucleotide sites in the L, M or both genes within a species (intra-L/M polymorphism). We then categorized these sites into non-synonymous and synonymous sites in the exons and intron sites. As shown in Table 3, the relative amount of divergence to polymorphism was significantly smaller in introns than in exon synonymous sites in all of the four species examined (*P*<0.02, Fisher's exact test). On the other hand, between non-synonymous and synonymous sites within exons, there was no significant difference detected in the relative amount of divergence to polymorphism (data not presented).

**Table 3:** Comparison of inter-LM divergence and intra-L/M polymorphism between synonymous sites and introns in gibbons

	No. of differences		
		
	Synonymous sites	Introns	Fisher's exact test (*P*)
*H. agilis *(L 10, M 10)			
Divergence	9	7	
Polymorphism	1	71	4.0 × 10^-7^

*H. lar *(L 10, M 10)			
Divergence	8	13	
Polymorphism	1	41	3.7 × 10^-4^

*H. pileatus *(L 9, M 9)			
Divergence	9	27	
Polymorphism	0	21	1.9 × 10^-2^

*S. syndactylus *(L 9, M 9)			
Divergence	9	4	
Polymorphism	1	26	2.3 × 10^-5^

### Among-group phylogenetic tree

**Figure 3:**
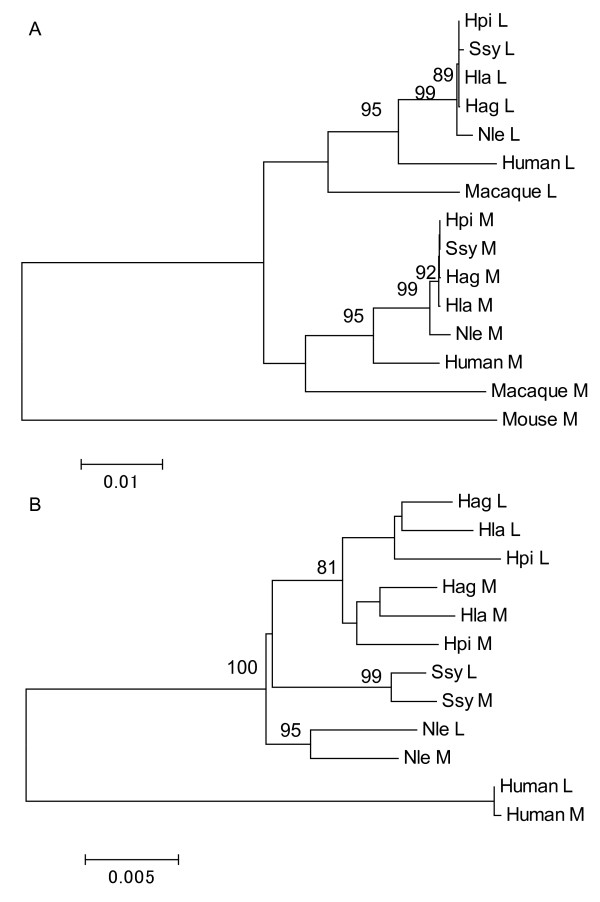
**The among-group phylogenetic trees of exons (A) and introns (B) of the L/M opsin genes of gibbons**. (A) The combined sequences of exons 3, 4 and 5 are considered. The human L and M, the crab-eating macaque L and M, and the mouse M opsin gene sequences are included. (B) The combined intron 3 and 4 sequences are considered. The human L and M opsin gene sequences are included. Bootstrap values over 80% are indicated at the branch nodes. Scale bars indicate the number of nucleotide substitution per site. Hag, *H. agilis*; Hla, *H. lar*; Hpi, *H. pileatus*; Nle, *N. leucogenys*; Ssy, *S. syndactylus*.

By combining the three exons and regarding one gene type (*i.e*. L or M opsin) in a species as a group, an among-group phylogenetic tree was constructed based on the nucleotide divergence between the groups (Figure 3A). The L and M opsin genes were separated at the common ancestor of the human, gibbons and macaque. In contrast, when the two introns were combined and an among-group phylogenetic tree was constructed for gibbons and human, the L and M opsin groups were clustered in *N. leucogenys*, *S. syndactylus*, human, and in the genus *Hylobates *(Figure 3B). In both the exon and intron trees, *Nomascus *was placed at the most basal position among the gibbons (Figure 3), being consistent with the current knowledge of the gibbon molecular phylogeny [53-57].

### Test of gene conversion by GENECONV

**Figure 4:**
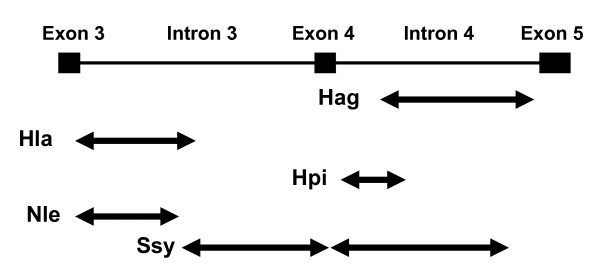
**Distribution of gene conversions between the L and M opsin genes in gibbons detected by GENECONV**. The top line represents the exon-intron structure of the L/M opsin genes. The arrows represent the area covered by gene conversions detected for pairs of L and M opsin genes in the five gibbon species (see Figure 3 legend for the abbreviations of species names).

For the GENECONV analysis we considered only male samples including the one *N. leucogenys *sample with single L and M opsin genes. We detected tracts of the gene conversion in each of the five species predominantly in intron regions with the global *P *values of less than 0.05 based on permutations for the entire alignment of the 78 sequences and corrected for multiple comparisons (Figure 4).

## Discussion

In the present study we examined intraspecific genetic variation of gibbon L and M opsin genes. This study also enabled comparison of the intraspecific nucleotide diversity between the L/M opsin gene regions and other autosomal regions as neutral references. In theory, when mutations are selectively neutral and population size is constant through generations, the nucleotide diversity is expected to be equal to the population mutation rate (4*N_e_*μ for autosomal and 3 *N_e_*μ for X-chromosomal genes of diploid organisms, where *N_e _*is the effective population size and μ is the mutation rate per nucleotide site per chromosome per generation) [58]. The nucleotide diversity of the gibbon neutral references was in the range of 0.10~0.41% (0.28% on average) when the two references were combined (Additional file 1, Table S5). These values are comparable to the nucleotide diversity of gibbon genomes recently reported by Kim et al. (2011) [52]; 0.16~0.29% (0.25% on average) in the autosomal regions and 0.04~0.18% (0.11% on average) in the X chromosomal regions of the same five species examined in this study (Figure 2 and Additional file 1, Table S5). Consistent with Kim et al. (2011) [52], they are also higher overall than reported nucleotide diversity of autosomal genes in humans (0.12%), chimpanzees (0.19%), bonobos (0.10%) and gorillas (0.15%) and are comparable with that in orangutans (0.36%) [59-61], suggesting a generally larger *N_e _*in Asian apes than in African apes. Although, our samples were collected from zoos and privately owned animals, the consistency with the large genome data set [52] implies that these samples could fairly represent the diversity of a natural population. The high nucleotide diversity of the reference genes also safely precludes the possibility that our samples are biased to a close kin group.

All 152 individuals from the eight species examined had both of the normal L and M opsin genes. Based on the composition of the three amino acid sites relevant to absorption spectra, there was no L/M hybrid gene detected with an intermediate spectral sensitivity. This observation is consistent with previous studies on the genetic variation of the L and M opsin genes in non-human catarrhines reporting no or rare observation of the gene deletion or the hybrid genes [19-21].

Ninety four individuals from five species, including 51 males, were subjected to sequencing of the 3.6~3.9-kb region encompassing exon 3 through exon 5. Among them, plural M opsin genes were detected in 10-80% of males in each species (23.5% of all males). Only a single L opsin sequence was detected in all males. Although more data are desired, these results are comparable to humans where multiple M copies are found in 66% of males of European origin [11] and 56% of Japanese males [62] and more than one L copy is rarely found [63]. Regarding non-human catarrhines, studies concur that a single L opsin copy is generally found in the genomes whereas some studies report that multiple M copies are rare [19,21,23,25] yet other studies report that they are common [26,29]. Thus, among Old World monkeys and apes, there seems to be no clear trend on the copy number variation of M opsin gene. There also seems to be no clear correlation between the frequency of X chromosomes carrying multiple copies of the M opsin gene with and the frequency of L or M opsin gene deletion or the hybridization.

In this study, we defined the L and M opsin genes on the basis of their deduced amino acid composition at residues 277 and 285 and not on the basis of their position in the L/M opsin gene array on the chromosome. As explained in the Methods, the sequence numbers of the L and M opsin gene groups could be underrepresented and the nucleotide diversity and divergence values for the gene groups could therefore be overestimated. Conceptually, the gene group in this study does not necessarily correspond to the "orthologous" group because it allows inclusion of duplicated copies. Nevertheless, when we only included the male samples with single L and M opsin genes to confine the analysis to the orthologous gene groups, the pattern of nucleotide divergence and diversity was indistinguishable from that where all samples were used (compare Figure 1 with Additional file 1, Figure S1, and Figure 2 with Additional file 1, Figure S2). Thus, there appears to be little or no distortion made in the pattern of nucleotide diversity and divergence by our collective treatment of the gene groups.

The within-species nucleotide divergence between L and M opsin genes was significantly higher in the exons than in the introns (Figure 1 and Additional file 1, Figure S1 and Table S2). This is opposite to what is expected for the general pattern of divergence: *i.e*. the protein-coding exons are generally more conservative than introns. Within the exons, however, non-synonymous divergence was not significantly higher than synonymous divergence in any of the three exons in the five species (Figure 1 and Additional file 1, Figure S1 and Table S3). This is an orthodox pattern of nucleotide divergence in coding regions under a functional constraint [64]. It is hence unlikely that the higher divergence in exons than in introns is due to divergent natural selection between the two genes acting on the amino acid sequence. The intron divergence was significantly lower than both of the synonymous and non-synonymous divergences in all of the five species (Figure 1 and Additional file 1, Figure S1). The low divergence in introns is likely due to a homogenizing process operating between the two genes, *i.e*. gene conversion, and the purifying selection against the gene conversion in the exons.

Contrary to the pattern of nucleotide divergence, in both L and M opsin genes the within-species nucleotide diversity was lower overall in exons than in introns (Figure 2 and Additional file 1, Figure S2 and Table S4). Within the exons, the variation was largely synonymous (Figure S3 and Additional file 1, Table S6). This is a typical pattern of nucleotide variation in protein-coding exons and introns. The nucleotide diversity of the introns was about the same magnitude with the nucleotide diversity of the autosomal neutral references multiplied by 3/4 and that of the X-chromosomal references in each species (Figure 2 and Additional file 1, Figure S2 and Table S5). Therefore, gene conversion in the introns does not seem to affect the nucleotide diversity, possibly because the numbers of introduced and erased mutations by the gene conversion are canceled out.

The contrasting pattern of the nucleotide divergence and diversity between exons and introns was further manifested by comparing the relative amount of the inter-LM divergence to the intra-L/M polymorphism between the synonymous sites and the introns. The ratio in the introns was significantly lower than that in the synonymous sites (Table 3). Since the synonymous sites and introns are both expected to be under selective neutrality, this pattern is consistent with the homogenization between the L and M opsin genes by gene conversion in introns. Within the exons, the ratio was not significantly different between synonymous and non-synonymous sites: in both nucleotide divergence and diversity the synonymous variation was generally larger than the non-synonymous variation (Figure 1 and Additional file 1, Figures S1 and S3). This suggests that the non-synonymous sites are under selective constraint and the non-synonymous differences between the two genes are protected by purifying selection in conjunction with neighboring synonymous differences from the homogenization by exon gene conversions.

The among-group phylogenetic tree for the L and M opsin genes showed a contrasting cluster pattern between cases using exon versus intron regions (Figure 3). In the exon tree, gene duplication appears to have occurred once in the common ancestor of hominoids (gibbons and human) and Old World monkeys (crab-eating macaque) (Figure 3A), whereas in the intron tree, gene duplication appears to have occurred independently in *N. leucogenys*, *S. syndactylus*, a common ancestor of the *Hylobates *species, and humans (Figure 3B). If the phylogenetic representation of the exon tree reflects the true relationships among the gene groups, the topology of the intron tree can be explained by the gene conversion between the duplicated L and M opsin genes at the individual level and by natural selection against the gene conversion involving exons at the population level. On the other hand, if the intron tree reflects the true relationships, the topology of the exon tree is inexplicable.

Tracts of gene conversions were assessed by the GENECONV statistical method (Figure 4). This method assesses whether the nucleotide differences found between two sequences are randomly distributed along the length of these sequences; it cannot detect gene conversions if a given region has fewer differences than its flanking regions. It has been advised that other methods, such as phylogenetic trees, have to be used to identify gene conversions (*e.g*. see [65]). Our GENECONV result is regarded consistent with the other results since the detected gene conversions were located mostly in the intron areas (Figure 4). In *S. syndactylus*, the detected gene conversion included the most part of the exon 4 (Figure 4). Considering that exon 4 contributes much less to the spectral difference between the L and M opsins than the exons 3 and 5 [5] and that the nucleotide divergence between the L and M opsin genes in the exon 4 is smaller than those in the exons 3 and 5 (Figure 1 and Additional file 1, Figure S1), the GENECONV result suggest that the purifying selection against gene conversion is weaker in the exons 4 than in the exons 3 and 5.

Throughout this study, we found no signature of positive (either directional or balancing) selection acting on the gibbon L and M opsin genes. As shown in Figure 1 and Additional file 1, Figure S1, the nucleotide divergence between the L and M opsin genes at non-synonymous sites was always smaller than that at synonymous sites. The nucleotide divergence between species for the L opsin genes or the M opsin genes at non-synonymous sites was also smaller or not significantly higher than that at synonymous sites (data not presented). Using the male samples with single L and M opsin genes (*i.e*. the "orthologous" sequence groups of the L and M opsin genes) of *H. agilis*, *H. lar*, *H. pileatus *and *S. syndactylus*, we detected no significant difference in the relative amount of the inter-specific divergence to the intra-specific polymorphism between synonymous and non-synonymous sites in either the L or M opsin gene (the McDonald-Kreitman test [66]) or between the L or M opsin gene and the neutral references (the HKA test [67]) (data not presented). Using these male samples, we did not find any characteristic haplotype structure in terms of linkage disequilibrium (LD) in the approximately 3.6~3.9-kb genomic region of the L and M opsin genes as was not found in a study of chimpanzee L opsin gene [25]. A measure of LD, the D', which takes a range from 0 to 1 [58], was nearly 1 throughout the region in all the four species examined (data not presented). This indicates low incidence of recombination between alleles, which is not unusual within such a short region. While the high LD could simply result from a small sample size, it is not contradictory with the frequent incidence of the gene conversion between gene loci because the LD concerns recombination between alleles.

The present study of within-species nucleotide variation of both the L and M opsin genes in gibbons provides additional support to previous studies suggesting that the gene conversion has homogenized the tandemly duplicated L and M opsin genes and their spectral difference is maintained by purifying selection on centrally-located exons in non-human catarrhines [13,14,26-29]. The strict conservation of trichromacy in non-human catarrhines underscores a general belief that trichromacy is selectively more advantageous than dichromacy or anomalous trichromacy. Primate trichromacy is hypothesized to be adaptive for detecting anything differing from the background foliage in red-green coloration, the proposed major objects being mature fruits, young leaves, pelage and skin [68-79].

The strict conservation of the trichromacy in non-human catarrhines is in sharp contrast to New World monkeys. In many species of New World monkeys, the spectral variation of the L/M opsin is achieved by an allelic differentiation of a single locus on the X chromosome. This results in polymorphic color vision, consisting of trichromacy in females and dichromacy in both sexes [80]. Genetic studies have shown that the allelic difference has been maintained by balancing selection [81-83]. It remains to be elucidated whether the polymorphic color vision is a consequence of selective advantage on trichromats through the overdominance of the L/M opsin heterozygotes or the consequence of other mechanisms of balancing selection including a mutual benefit among different vision types [83,84]. It has been demonstrated that dichromats are superior to trichromats in some visual tasks such as defeating cryptic coloration and motion detection [70,85,86] and have a foraging advantage for surface-dwelling insects [87-89]. In non-human catarrhines, however, this benefit of dichromacy could have been overwhelmed by that of trichromacy. It is still an open question whether the difference between the non-human catarrhines (routine trichromacy) and New World monkeys (polymorphic color vision) is attributable to a phenological difference among continents, such as in severity of seasonality and availability of non-colorful and non-seasonal fig and palms [90], to a dietary variation such as in dependency on insets or colorful fruits, or to variation of social color signals [78,79].

The absence of L/M defects in gibbons and other non-human catarrhines reveals the uniqueness of human color vision, in which the deletion of L or M opsin genes and the presence of L/M hybrid genes causes relatively high incidence of dichromacy and anomalous trichromacy [18,91-94]. Color vision polymorphisms among humans could be explained by frequent gene conversions of exon 3, due to the presence of a recombination hot-spot chi element conserved in the exon [17] and relaxation of the selective constraint to maintain the spectral difference between the L and M opsin genes. Alternatively, adaptive explanations are possible. Balancing selection may be acting on subtle changes in light absorption of L [18] and M opsins among females, or a selective advantage could be experienced by dichromatic males during hunting activities or via an increased ability to detect predators via penetrating camouflage [85]. Further population studies of the L/M opsin genes for terrestrial-savanna catarrhines, such as baboons, would be important for elucidating evolutionary forces acting on development and maintenance of primate trichromacy.

## Conclusions

The present study of within-species nucleotide variation of the L and M opsin genes in gibbons supports the notion that gene conversion has homogenized the tandemly duplicated L and M opsin genes and that their spectral difference is maintained by the purifying selection on the centrally-located exons in non-human catarrhines. Growing knowledge on genetic variation of opsin genes and visual ecology of primates has led to a paradigm shift toward the view that the adaptive value of trichromacy is conditional rather than universal, depending on the specific ecological demands on animals in their environments. Further population genetic and molecular phylogenic studies of primate visual opsins including humans, as well as observation and experiments on vision-guided primate behaviors, and phenological studies of habitats of wild primates will elucidate the detailed conditions of color vision evolution in primates.

## Authors' contributions

SK and AM conceived of the study. SK designed and supervised the experiments and analyses. MK organized the sample collection and DNA extraction. BSu organized the sample collection in Indonesia. DP conducted DNA extractions in Indonesia. SM organized the sample collection and conducted DNA extractions in Thailand. BSi organized the sample collection in Thailand. SG conducted the veterinary care of the animals during the sampling. TH carried out experiments, data compiling, and analysis. SK and TK performed additional data analyses. SK and TH wrote and illustrated the manuscript. HO advised on the research design and manuscript preparation. All authors read and approved the final manuscript.

## Supplementary Material

Additional file 1 **Supplementary tables and figures**. Tables S1, S2, S3, S4, S5 and S6 and Figures S1, S2 and S3. Click here for file

Additional file 2 **Alignment of the gibbon L and M opsin gene exon 3**. A "sequential" (fasta) format of the sequence alignments for the gibbon L and M opsin gene exon 3. Click here for file

Additional file 3 **Alignment of the gibbon L and M opsin gene intron 3**. A "sequential" (fasta) format of the sequence alignments for the gibbon L and M opsin gene intron 3. Click here for file

Additional file 4 **Alignment of the gibbon L and M opsin gene exon 4**. A "sequential" (fasta) format of the sequence alignments for the gibbon L and M opsin gene exon 4. Click here for file

Additional file 5 **Alignment of the gibbon L and M opsin gene intron 4**. A "sequential" (fasta) format of the sequence alignments for the gibbon L and M opsin gene intron 4. Click here for file

Additional file 6 **Alignment of the gibbon L and M opsin gene exon 5**. A "sequential" (fasta) format of the sequence alignments for the gibbon L and M opsin gene exon 5. Click here for file

Additional file 7 **Alignment of the gibbon eta-globin pseudogene 5' flanking region**. A "sequential" (fasta) format of the sequence alignments for the gibbon eta-globin pseudogene 5' flanking region. Click here for file

Additional file 8 **Alignment of the gibbon S opsin gene intron 4**. A "sequential" (fasta) format of the sequence alignments for the gibbon S opsin gene intron 4. Click here for file
