# Supplementary material for: Gene conversion and purifying selection shape nucleotide variation in gibbon L/M opsin genes
Source: BMC Evol Biol. 2011 Oct 22;11:312. doi: 10.1186/1471-2148-11-312 (PMC3213168; doi:10.1186/1471-2148-11-312)
Supplement: Additional file 1 — Supplementary tables and figures. Tables S1, S2, S3, S4, S5 and S6 and Figures S1, S2 and S3. [file 1471-2148-11-312-S1.PDF]

Table S1 The number of gibbon individuals for which the L and M opsin genes were genotyped

Species name	Male	Female	Total
Hylobate agilis	18	17	35
H. klossii	1	1	2
H. lar	16	23	39
H. moloch	2	4	6
H. muelleri	3	3	6
H. pileatus	12	7	19
Nomascus leucogenys	6	10	16
Symphalangus syndactylus	14	15	29
Total	72	80	152

Table S2 Nucleotide divergence between L and M opsin genes in gibbons

		Hag	Hla	Hpi	Nle	Ssy
	No. of sequence	L 28, M 30	L 34, M 38	L 22, M 23	L 21, M 25	L 32, M 33
Exon 3	(bp)	169	169	169	169	169
	d (x 10^{-2})	4.73	4.69	4.73	4.73	4.83
	SE of *d* (x 10^{-2})	1.64	1.55	1.58	1.50	1.58
Exon 4	(bp)	166	166	166	166	166
	d (x 10^{-2})	2.41	2.41	2.41	2.53	2.41
	SE of *d* (x 10^{-2})	1.19	1.18	1.18	1.16	1.19
Exon 5	(bp)	240	240	240	240	240
	d (x 10^{-2})	5.03	5.00	5.00	5.06	5.07
	SE of *d* (x 10^{-2})	1.37	1.37	1.37	1.34	1.36
Exon total	(bp)	575	575	575	575	575
	d (x 10^{-2})	4.19	4.16	4.17	4.23	4.23
	SE of *d* (x 10^{-2})	0.78	0.78	0.78	0.83	0.78
Intron 3	(bp)	1455	1467	1464	1465	1469
	d (x 10^{-2})	1.03	1.12	1.56	0.61	0.56
	SE of *d* (x 10^{-2})	0.24	0.24	0.29	0.16	0.16
Intron 4	(bp)	1544	1523	1556	1557	1556
	d (x 10^{-2})	1.00	1.27	1.08	1.24	0.39
	SE of *d* (x 10^{-2})	0.21	0.28	0.24	0.25	0.13
Intron total	(bp)	2999	2990	3020	3022	3025
	d (x 10^{-2})	1.02	1.19	1.31	0.93	0.47
	SE of *d* (x 10^{-2})	0.16	0.18	0.21	0.16	0.10

Hag: *Hylobates agilis*

Hla: *Hylobates lar*

Hpi: *Hylobates pileatus*

Nle: *Nomascus leucogenys*

Ssy: *Symphalangus syndactylus*

Table S3 Synonymous (S) and non-synonymous (N) nucleotide divergence between L and M opsin genes in gibbons

	Hag		Hla		Hpi		Nle		Ssy	
	S	N	S	N	S	N	S	N	S	N
Exon 3										
d (x 10^{-2})	6.51	4.35	6.23	4.37	6.51	4.35	6.51	4.35	6.91	4.35
SE of *d* (x 10^{-2})	3.94	1.91	3.76	1.92	3.87	1.83	3.79	1.93	3.97	1.88
Exon 4										
d (x 10^{-2})	2.34	2.45	2.34	2.45	2.34	2.45	2.81	2.45	2.34	2.45
SE of *d* (x 10^{-2})	2.20	1.76	2.36	1.81	2.24	1.74	2.37	1.86	2.37	1.78
Exon 5										
d (x 10^{-2})	9.02	3.82	8.96	3.80	8.96	3.80	9.21	3.80	9.02	3.87
SE of *d* (x 10^{-2})	3.77	1.38	3.85	1.35	3.91	1.32	3.71	1.35	3.68	1.37
Exon total										
d (x 10^{-2})	6.20	3.57	6.10	3.57	6.18	3.56	6.43	3.56	6.32	3.59
SE of *d* (x 10^{-2})	1.89	0.92	2.01	0.93	1.93	0.91	1.90	0.91	1.95	0.95

Table S4 Nucleotide diversity of L and M opsin gene regions in gibbons

		Hag		Hla		Hpi		Nle		Ssy	
		L	M	L	M	L	M	L	M	L	M
	No. of sequence	28	30	34	38	22	23	21	25	32	33
Exon 3	(bp)	169	169	169	169	169	169	169	169	169	169
	π (x 10^{-2})	0	0	0	0.146	0	0	0	0	0.161	0
	SE of π (x 10^{-2})	0	0	0	0.118	0	0	0	0	0.156	0
Exon 4	(bp)	166	166	166	166	166	166	166	166	166	166
	π (x 10^{-2})	0	0	0	0	0	0	0.155	0.092	0	0
	SE of π (x 10^{-2})	0	0	0	0	0	0	0.149	0.089	0	0
Exon 5	(bp)	240	240	240	240	240	240	240	240	240	240
	π (x 10^{-2})	0.030	0.084	0	0	0	0	0.115	0	0.123	0
	SE of π (x 10^{-2})	0.029	0.058	0	0	0	0	0.086	0	0.085	0
Exon total	(bp)	575	575	575	575	575	575	575	575	575	575
	π (x 10^{-2})	0.012	0.034	0	0.043	0	0	0.093	0.027	0.099	0
	SE of π (x 10^{-2})	0.012	0.023	0	0.034	0	0	0.055	0.026	0.058	0
Intron 3	(bp)	1751	1455	1775	1467	1773	1469	1468	1465	1470	1487
	π (x 10^{-2})	0.266	0.380	0.130	0.176	0.205	0.020	0.381	0.323	0.276	0.067
	SE of π (x 10^{-2})	0.064	0.077	0.041	0.054	0.060	0.020	0.095	0.095	0.086	0.021
Intron 4	(bp)	1555	1545	1524	1556	1556	1558	1557	1558	1556	1558
	π (x 10^{-2})	0.370	0.313	0.188	0.118	0.022	0.213	0.295	0.273	0.149	0.089
	SE of π (x 10^{-2})	0.088	0.061	0.044	0.033	0.017	0.068	0.089	0.084	0.057	0.038
Intron total	(bp)	3306	3000	3299	3023	3329	3027	3025	3023	3026	3045
	π (x 10^{-2})	0.315	0.346	0.156	0.146	0.119	0.119	0.337	0.297	0.211	0.078
	SE of π (x 10^{-2})	0.046	0.051	0.033	0.032	0.033	0.032	0.065	0.063	0.050	0.021

Table S5 Nucleotide diversity of the neutral reference regions in gibbons

		Hag	Hla	Hpi	Nle	Ssy
Eta globin						
	No. of sequence	60	78	36	32	62
	(bp)	563	618	622	576	522
	π (x 10^{-2})	0.568	0.304	0.138	0.760	0.385
	SE of π (x 10^{-2})	0.146	0.097	0.079	0.179	0.101
	3/4 x π (x 10^{-2})	0.426	0.228	0.104	0.570	0.289
	3/4 x SE of π (x 10^{-2})	0.110	0.073	0.059	0.134	0.076
S opsin						
	No. of sequence	70	72	32	28	58
	(bp)	565	503	529	500	519
	π (x 10^{-2})	0.281	0.082	0.046	0.055	0.257
	SE of π (x 10^{-2})	0.129	0.048	0.029	0.036	0.099
	3/4 x π (x 10^{-2})	0.211	0.062	0.035	0.041	0.193
	3/4 x SE of π (x 10^{-2})	0.097	0.036	0.022	0.027	0.074
Neutral total						
	No. of sequence	56	72	32	28	58
	(bp)	1128	1121	1151	1076	1041
	π (x 10^{-2})	0.413	0.188	0.102	0.360	0.327
	SE of π (x 10^{-2})	0.097	0.056	0.050	0.087	0.079
	3/4 x π (x 10^{-2})	0.310	0.141	0.077	0.270	0.245
	3/4 x SE of π (x 10^{-2})	0.073	0.042	0.038	0.065	0.059
Kim et al. (2011)						
	No. of samples	3	1	2	7	1
Autosomal						
	Length (bp)	29872	36620	41740	75078	33198
	π (x 10^{-2})	0.29	0.24	0.29	0.29	0.16
	3/4 x π (x 10^{-2})	0.22	0.18	0.22	0.22	0.12
X chromosomal						
	Length (bp)	2341	NE	5937	13453	7068
	π (x 10^{-2})	0.18	NE	0.04	0.13	0.10

Table S6 Synonymous and nonsynonymous nucleotide diversity of the L/M opsin exons in gibbons

	Hag				Hla				Hpi				Nle				Ssy			
	L		M		L		M		L		M		L		M		L		M	
Exon 3	S	N	S	N	S	N	S	N	S	N	S	N	S	N	S	N	S	N	S	N
π (x 10^{-2})	0	0	0	0	0	0	0.508	0.041	0	0	0	0	0	0	0	0	0.704	0	0	0
SE of π (x 10^{-2})	0	0	0	0	0	0	0.516	0.041	0	0	0	0	0	0	0	0	0.722	0	0	0
Exon 4																				
π (x 10^{-2})	0	0	0	0	0	0	0	0	0	0	0	0	0.600	0	0.361	0	0	0	0	0
SE of π (x 10^{-2})	0	0	0	0	0	0	0	0	0	0	0	0	0.611	0	0.367	0	0	0	0	0
Exon 5																				
π (x 10^{-2})	0	0.039	0.364	0	0	0	0	0	0	0	0	0	0.492	0	0	0	0	0.161	0	0
SE of π (x 10^{-2})	0	0.037	0.259	0	0	0	0	0	0	0	0	0	0.343	0	0	0	0	0.113	0	0
Exon total																				
π (x 10^{-2})	0	0.016	0.143	0	0	0	0.141	0.012	0	0	0	0	0.386	0	0.112	0	0.197	0.068	0	0
SE of π (x 10^{-2})	0	0.016	0.105	0	0	0	0.138	0.012	0	0	0	0	0.226	0	0.107	0	0.191	0.047	0	0

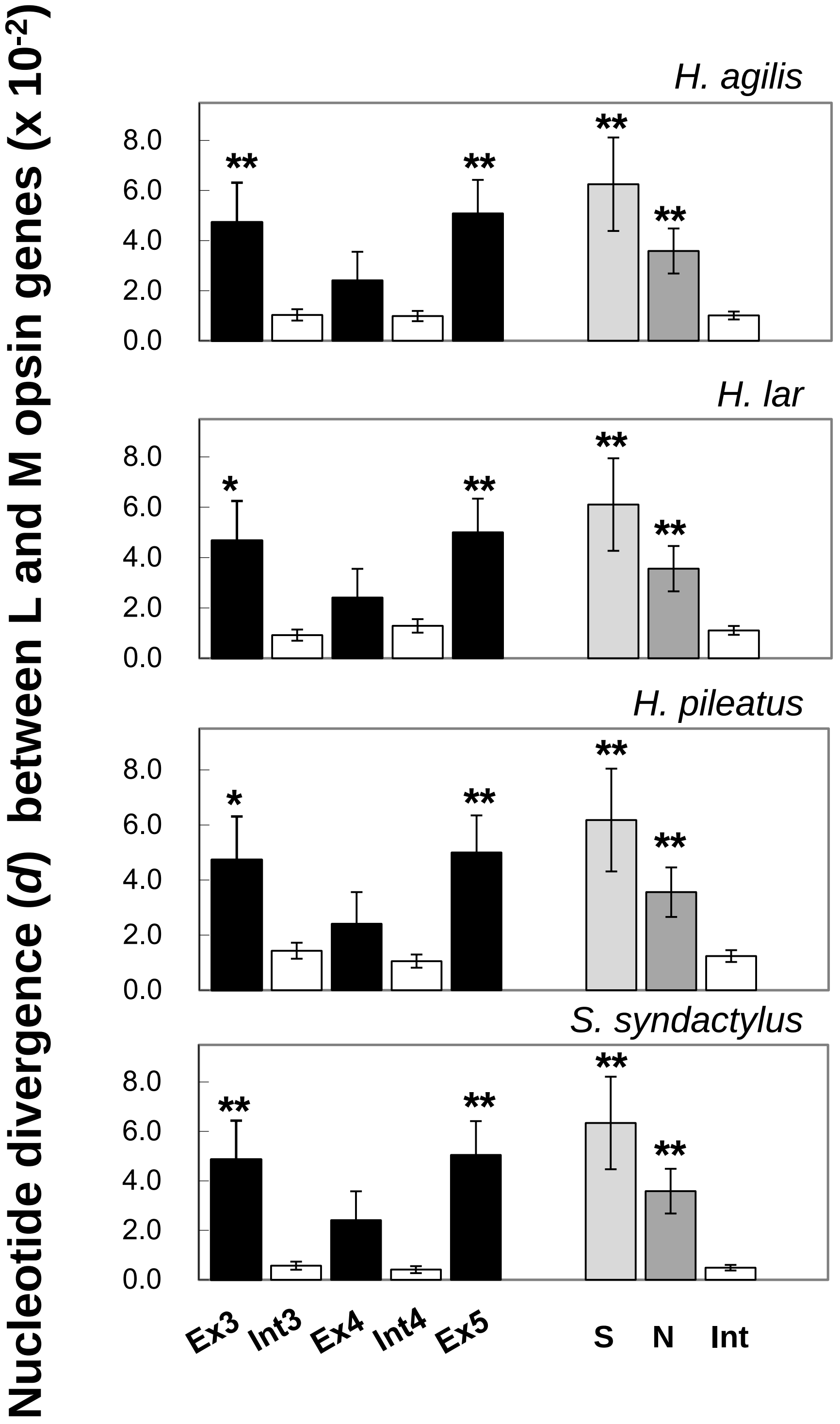
Nucleotide divergence (d) between L and M opsin genes (x 10-2)
H. agilis
H. lar
H. pileatus
S. syndactylus
8.0
6.0
4.0
2.0
0.0
*
**
Ex3
Int3
Ex4
Int4
Ex5
S
N
Int

Figure S1

Figure S1 The nucleotide divergence (*d*) between the L and M opsin genes in four species of gibbons, *H. agilis*, *H. lar*, *H. pileatus* and *S. syndactylus*. Only males with single L and M opsin genes are considered. The *d* values of the exons are represented by black, the introns by white, the synonymous sites by light gray, and the non-synonymous sites by dark gray bars. The error bars indicate the estimated standard error of the *d* values based on the 1000 x bootstrap resampling. The asterisks indicate that the *d* values are significantly higher than the *d* value of the combined sequence of the introns 3 and 4 in each species. The single and double asterisks represent the statistical significance at 0.05 and 0.01 levels, respectively, based on the one-tailed Z test. Ex3, exon 3; Ex4, exon 4; Ex5, exon 5, Int3, intron 3; Int4, intron 4; Int, the introns 3 and 4 combined; S, synonymous sites in the exons 3, 4 and 5; N, non-synonymous sites in the exons 3, 4 and 5.

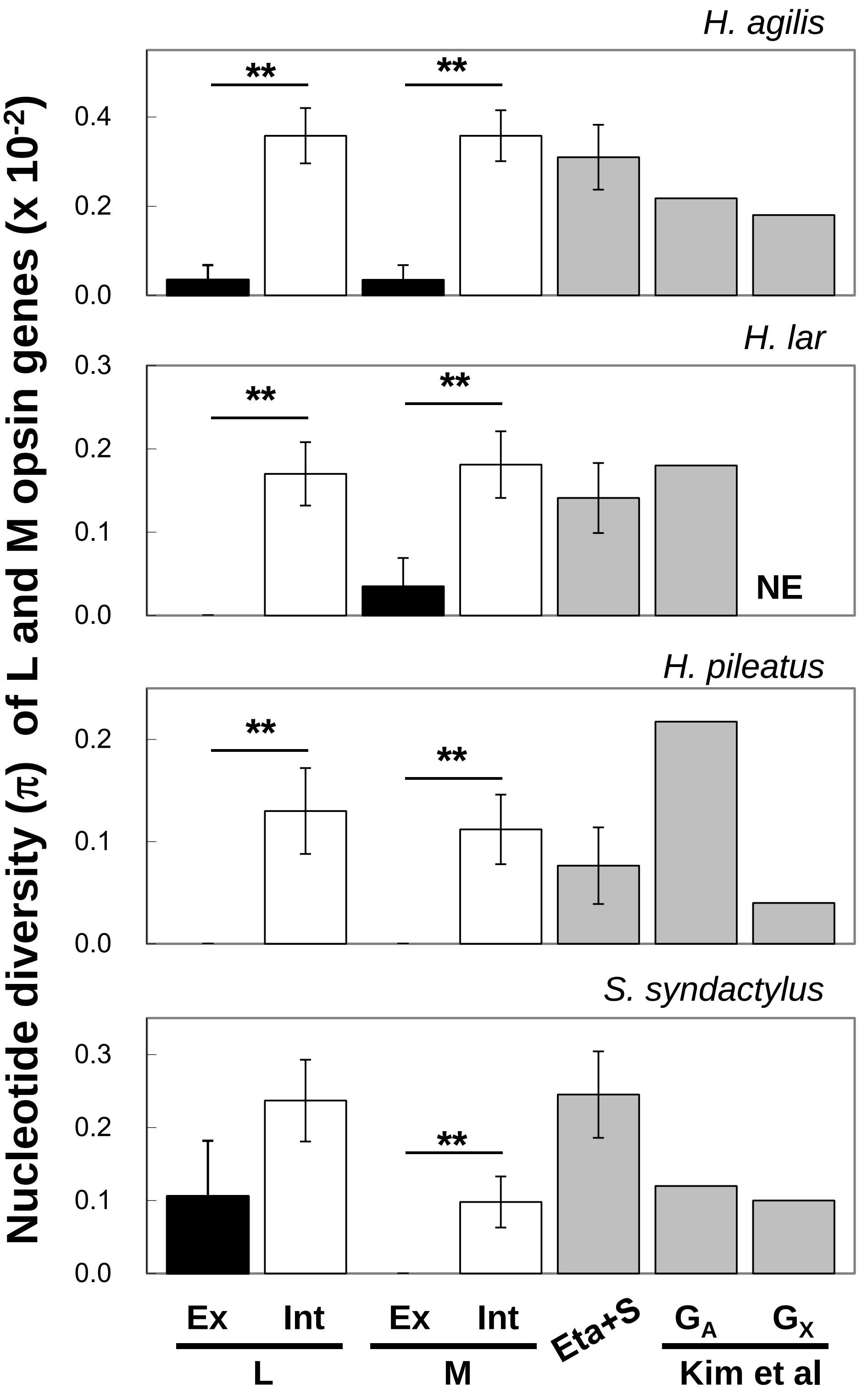

H. agilis
H. lar
H. pileatus
S. syndactylus
Nucleotide diversity (π) of L and M opsin genes (x 10-2)
**
NE
0.0
0.1
0.2
0.3
0.4
Ex
Int
Ex
Int
Eta+S
GA
GX
L
M
Kim et al

Figure S2

Figure S2 The nucleotide diversity (π) of the exons (black bar) and the introns (white bar) of the L and M opsin genes and the neutral references (gray bars) in the four species of gibbons. Only males with single L and M opsin genes are considered. The gibbon genomic data reported [52] for autosomal (G_A) and X-chromosomal (G_X) regions are also indicated as neutral references. The π values of the combined sequences of the eta globin pseudogene and the S opsin intron 4 (Eta+S) and the π values of G_A are multiplied by 3/4. The single and double asterisks represent the statistical significance at 0.05 and 0.01 levels, respectively, based on the one-tailed Z test.

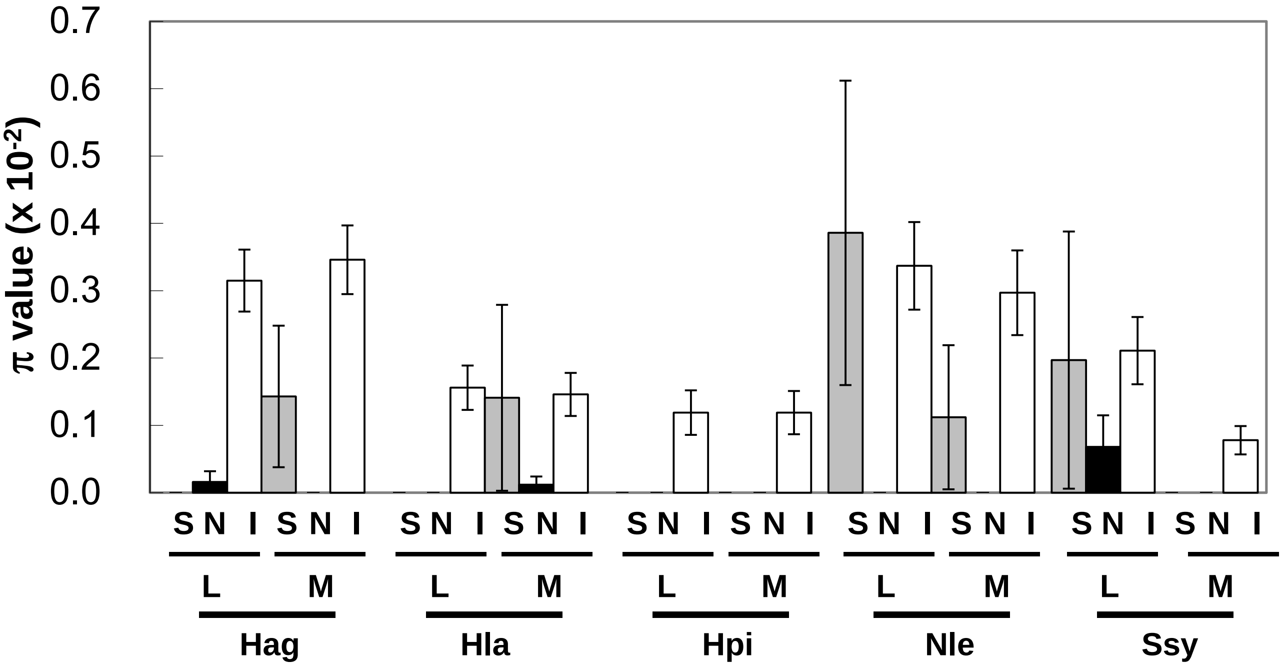
0.7
0.6
0.5
0.4
0.3
0.2
0.1
0.0
π value (x 10-2)
S N I S N I S N I S N I S N I S N I S N I S N I S N I S N I
L M L M L M L M L M
Hag
Hla
Hpi
Nle
Ssy

Figure S3

Figure S3 The synonymous (S; gray bar) and non-synonymous (N; black bar) nucleotide diversity (π) of the combined exons 3, 4 and 5 and the π value of the combined introns 3 and 4 (I, white bar) of the L and M opsin genes in the five species of gibbons. Hag, *H. agilis*; Hla, *H. lar*; Hpi, *H. pileatus*; Nle, *N. leucogenys*; Ssy, *S. syndactylus*.
